# A case of autonomous cortisol secretion in a patient with subclinical Cushing’s syndrome, *GNAS* mutation, and paradoxical cortisol response to dexamethasone

**DOI:** 10.1186/s12902-019-0345-8

**Published:** 2019-01-22

**Authors:** Chihiro Sakaguchi, Kenji Ashida, Kenichi Kohashi, Kenji Ohe, Yoichi Fujii, Seiichi Yano, Yayoi Matsuda, Shohei Sakamoto, Ryuichi Sakamoto, Yoshinao Oda, Masatoshi Nomura, Yoshihiro Ogawa

**Affiliations:** 1 Department of Medicine and Bioregulatory Science, Graduate School of Medical Sciences, Kyushu University, Fukuoka, Japan; 2 Division of Endocrinology and Metabolism, Department of Internal Medicine, Kurume University School of Medicine, 67 Asahi-machi, Kurume-city, Fukuoka, 830-0011 Japan; 3 Department of Anatomic Pathology, Pathological Sciences, Graduate School of Medical Sciences, Kyushu University, Fukuoka, Japan; 4 Faculty of Pharmaceutical Sciences, Fukuoka University, Fukuoka, Japan; 5 Department of Pathology and Tumor Biology, Graduate School of Medicine, Kyoto University, Kyoto, Japan

**Keywords:** Cushing’s syndrome, GNAS, Adrenal tumor, Cortisol, Cyclic-AMP, Protein kinase A

## Abstract

**Background:**

Increased urinary free cortisol in response to the oral administration of dexamethasone is a paradoxical reaction mainly reported in patients with primary pigmented nodular adrenocortical disease. Here, we describe the first case of subclinical Cushing’s syndrome represented by autonomous cortisol secretion and paradoxical response to oral dexamethasone administration, harboring an activating mutation in the α subunit of the stimulatory G protein *(GNAS)*.

**Case presentation:**

A 65-year-old woman was diagnosed with subclinical Cushing’s syndrome during an evaluation for bilateral adrenal masses. Tumors of unknown origin were found in the heart, brain, thyroid gland, colon, pancreas, and both adrenal glands. Adenocarcinoma of the sigmoid colon and systemic brown-patchy skin pigmentation were also present. Her urinary cortisol levels increased in response to oral dexamethasone, while serum dehydroepiandrosterone-sulfate was not suppressed. After right adrenalectomy, genetic analysis of the resected tumor revealed the somatic *GNAS* activating mutation, p.R201H. Paradoxical urinary cortisol response persisted even after unilateral adrenal resection, although serum and urinary cortisol levels were attenuated.

**Conclusions:**

This patient harbored a *GNAS* activating mutation, and presented with a mild cortisol- and androgen-producing adrenal adenoma. Administration of oral dexamethasone paradoxically increased cortisol levels, possibly via the stimulation of the cyclic adenosine monophosphate-dependent protein kinase A signaling pathway, which is seen in patients with pigmented nodular adrenocortical disease or Carney complex. *GNAS* mutations may provide clues to the mechanisms of hyper-function and tumorigenesis in the adrenal cortex, especially in bilateral adrenal masses accompanied by multiple systemic tumors. Examining *GNAS* mutations could help physicians detect extra-adrenal malignancies, which may contribute to an improved prognosis for patients with this type of Cushing’s syndrome.

## Background

The cyclic adenosine monophosphate (cAMP)-dependent protein kinase A (PKA) signaling pathway is considered a mainstay for adrenal steroid production and cell proliferation. Several mutations in the cAMP-PKA pathway are responsible for autonomous production of glucocorticoids and adrenal tumorigenesis, resulting in Cushing’s syndrome [1–5].

Inactivating mutation of the cAMP-dependent protein kinase type 1α regulatory subunit (*PRKAR1A*) is responsible for both primary pigmented nodular adrenocortical disease (PPNAD) and the Carney complex [6]. Paradoxical cortisol response to dexamethasone administration is a unique response observed in 69% of PPNAD cases [7]. However, few reports have described this response [8, 9], and the underlying mechanisms remain unclear.

We describe a case of subclinical Cushing’s syndrome (SCS) [10] with autonomous cortisol secreting adrenal tumor [11], who showed this paradoxical reaction due to harboring an activating mutation in the α subunit of the somatic stimulatory G protein *(GNAS)*. Although *GNAS* mutations have been well-described in the McCune Albright syndrome [4], sporadic mutations of this gene have been reported in various neoplasms [12], including corticosteroid-producing adrenal tumors that can lead to Cushing’s syndrome [1–5, 12]. The present case may confirm and show features of autonomous steroid production and adrenocortical tumorigenesis leading to SCS via constitutive activation of the cAMP-dependent PKA pathway.

## Case presentation

**Fig. 1:**
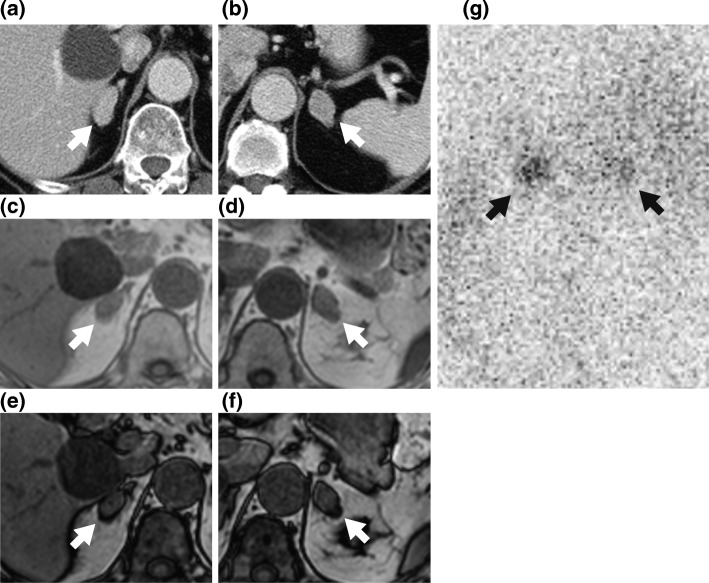
Imaging study of bilateral adrenal masses. Computed tomography scan shows bilateral adrenal tumors. (**a**) right side, 25 × 13 mm and (**b**) left side, 18 × 15 mm. Magnetic resonance imaging showed low intensity loci in adrenal tumors in “out phase” compared with “in phase” of chemical shift imaging on T1-weighted image. (**c**) right, in-phase; (**d**) right, out-phase; (**e**) left, in-phase; (**f**) left, out-phase. (**g**) ^131^I-adosterol scintigraphy shows bilateral adrenal accumulation. Arrows in each image designate the adrenal tumors or more accurately where the adosterol is accumulating in the adrenal tumors

**Fig. 2:**
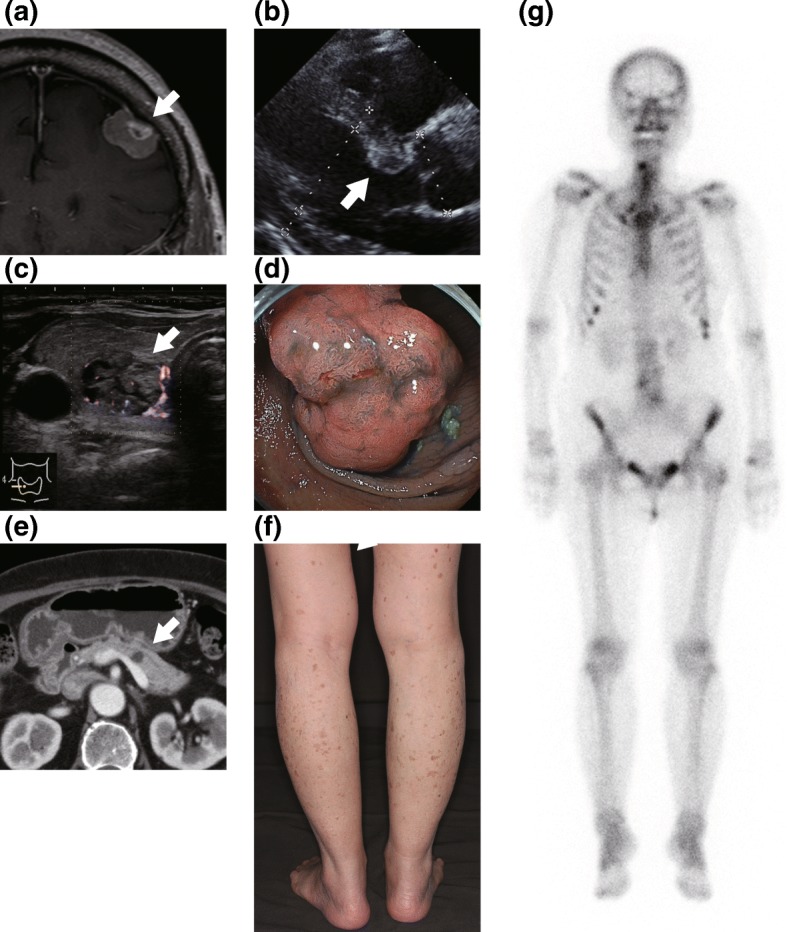
Imaging studies of extra-adrenal tumors in this patient. **a** A convexity meningioma 21 × 13 × 18 mm in size on enhanced T1WI MRI of the Head (arrow). The pituitary tumor is not marked. **b** A hyperechoic lesion about 14 mm in size in the interventricular septum on echocardiography (arrow). **c** Cervical ultra-sonography revealed four tumors in both thyroid lobes (arrow). **d** Colonoscopy shows tubular adenocarcinoma in the colon. This tumor was later resected, but the status of *GNAS* mutation was not assessed. **e** Enhanced abdominal computed tomography showed cystic lesions compatible with intraductal papillary mucinous neoplasm in the pancreatic body (arrow). **f** Brown patchy pigmentations were observed systemically. Skin appearance with patchy pigmentations of bilateral lower limbs is shown. **g** X-ray imaging and bone scintigraphy with ^99m^Tc-scintigraphy did not indicate fibrous dysplasia

A 65-year-old woman was referred to our endocrinology center for evaluation of diabetes mellitus, hyperlipidemia, and bilateral adrenal masses, which were detected for the first time prior to a scheduled operation for sigmoid-colon cancer. She previously underwent total hysterectomy for uterine fibroids at the age of 44. She was on anti-hypertensive medication from approximately 40 years of age, and had experienced aortic dissection at the age of 56. She showed normal stature and a body mass index of 24.7 kg/m^2^. She did not show any Cushingoid signs. Fasting morning serum cortisol and urinary free cortisol levels (measured by immune radio metric assay method, SRL, Tokyo) were normal (Table 1). However, midnight levels of serum cortisol were high, and both overnight dexamethasone suppression tests, using 1 mg and 8 mg dexamethasone, did not suppress serum cortisol or dehydroepiandrosterone (DHEA) -sulfate levels. Plasma ACTH levels were low and did not respond to 100 μg of intravenous corticotropin-releasing hormone. Furthermore, a dexamethasone suppression test using Liddle’s method [7] showed a paradoxical increase in the levels of urinary cortisol (Table 2). The ratio of plasma aldosterone concentration (PAC) to plasma renin activity (PRA) was significantly high, although PAC was within the normal range. Based on the results of endocrinological examinations, the patient was diagnosed with idiopathic hyperaldosteronism [13] (Tables 1 and 2). Adrenal venous sampling indicated bilateral aldosterone hypersecretion (Table 1). Bilateral adrenal tumors, 25 × 13 mm and 18 × 15 mm, in the right and left gland respectively, had the appearance of adrenocortical adenoma on computed tomography (Fig. 1a, b) and magnetic resonance imaging (Fig. 1c-f). Accumulations of ^131^I-adosterol in adrenal tumors were observed on both sides, though predominantly on the left (Fig. 1g). Various extra-adrenal masses were detected in several imaging modalities, and patchy brown skin pigmentations were observed systemically (Fig. 2).

**Table 1:** Laboratory data of the present patient

Variable, unit	Value	Reference range	Variable, unit	Value	Reference range
<Blood examination>			<Urine analysis>		
Fasting plasma glucose, mg/dL	111	73–109	Free cortisol, μg/day	73.5	11.2–80.3
Hemoglobin A_1_c, %	7.7	4.9–6.0	Androsterone, mg/day	0.31	0.4–3.00
Na, mmol/L	143	138–145	Etiocholanolone, mg/day	0.56	0.30–2.50
K, mmol/L	3.3	3.6–4.8	Dehydroepiandrosterone, mg/day	0.02	0.04–2.60
Cl, mmol/L	105	101–108	11-OH-Androsterone, mg/day	0.4	0.22–1.60
ACTH (0900 h), pg/mL	3.1	7.2–63.3	11-OH-Etiocholanorone, mg/day	0.27	0.02–0.65
ACTH (2300 h), pg/mL	2.5	N/A	11-keto-Androsterone, mg/day	0.01	< 0.07
Cortisol (0900 h), μg/dL	10.6	4.0–18.3	11-keto-Etiocholanolone, mg/day	0.1	0.03–0.50
Cortisol (2300 h), μg/dL	11.4	< 5.0			
Dehydroepiandrosterone sulfate, μg/dL	129	12–133	<Adrenal venous sampling>		
Total testosterone, ng/mL	0.26	0.11–0.47	Cortisol (rt. adrenal vein), μg/dL	361	
Corticosterone, ng/mL	2.36	0.12–8.48	Cortisol (lt. adrenal vein), μg/dL	380	
Deoxycorticosterone, ng/mL	0.09	0.03–0.33	Cortisol (inferior vena cava), μg/dL	33.6	
PRA, ng/mL/h	0.2	0.3–2.9	PAC (rt. adrenal vein), pg/mL	10,900	
PAC, pg/mL	88.3	29.9–159	PAC (lt. adrenal vein), pg/mL	13,000	
			PAC (inferior vena cava), pg/mL	328	

Adrenal venous sampling was performed while administering continuous intravenous infusion of ACTH (50 μg/hour). Text in parentheses indicate the location of blood sampling

ACTH, adrenocorticotropic hormone; PRA, plasma renin activity; PAC, plasma aldosterone concentration

**Table 2:** Endocrinological data of loading test

Loading agent	Variable, unit	Value, pre-operation	Value, post-operation	Reference range
1 mg of DEX, overnight	Serum cortisol, μg/dL	11.8	4.6	< 1.8
8 mg of DEX, overnight	Serum cortisol, μg/dL	13.1	8.7	< 1.0
100 μg of CRH intravenously	Basal ACTH, pg/mL	2.7	2.6	7.2–63.3
	Peak ACTH, pg/mL (time, min)	5.2 (90)	9.1 (90)	> 2× basal ACTH
2 L of saline intravenously	PAC (before loading), pg/mL	78	73	29.9–159
	PAC (after loading), pg/mL	92	81	< 60
50 mg of captopril orally	PAC (0 min), pg/mL	56	114	29.9–159
	PAC (60 min), pg/mL	59	102	N/A
	PAC (90 min), pg/mL	50	82	N/A
	ARR (0 min)	140	285	< 200
	ARR (60 min)	590	102	< 200
	ARR (90 min)	496	74	< 200
40 mg of furosemide intravenously with keeping upright	PRA (0 min), ng/mL/h	0.3	0.4	0.3–2.9
	PRA (60 min), ng/mL/h	0.3	0.5	> 2.0
	PRA (120 min), ng/mL/h	0.4	0.8	> 2.0
<Dexamethasone suppression test, Liddle’s method>				
No DEX	Urinary- free cortisol, μg/day	74	14	11.2–80.3
2 mg/day of DEX, the first day	Urinary- free cortisol, μg/day	538	32	
2 mg/day of DEX, the second day	Urinary- free cortisol, μg/day	284	28	
8 mg/day of DEX, the first day	Urinary- free cortisol, μg/day	141	21	
8 mg/day of DEX, the second day	Urinary- free cortisol, μg/day	136	20	

Overnight suppression test with 1 mg and 8 mg of DEX did not suppress cortisol levels, but paradoxically resulted in an increase in urinary cortisol levels

DEX, dexamethasone; CRH, corticotropin-releasing hormone; ACTH, adrenocorticotropic hormone; PAC, plasma aldosterone concentration; ARR, plasma aldosterone / plasma renin activity ratio; PRA, plasma renin activity; N/A, not applicable

**Fig. 3:**
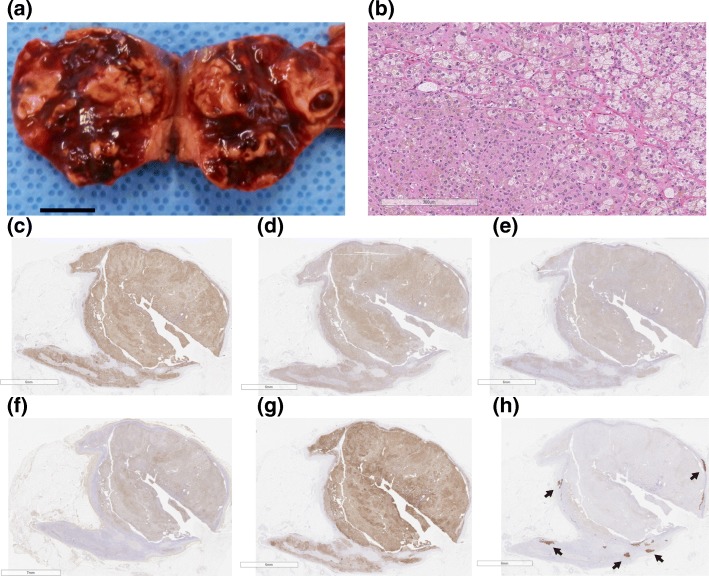
Histopathological examination of adrenal adenoma. **a** Macro-image of the resected adrenal adenoma. **b** Micro-image with low magnitude using hematoxylin-eosin staining. The adrenal adenoma composed of compact cells and clear cells. **c**-**h** Immunohistochemical staining was performed using anti-human antibodies as follows: **c** CYP17A1, **d** HSD3B type-1, **e** HSD3B type-2, **f** Dehydroepiandrosterone sulfotransferase, **g** CYP11B1, and **h** CYP11B2. The adrenal adenoma showed positive signal for cortisol producing enzymes and DHEA-sulfotransferase, which might be responsible for the high-normal serum level of DHEA-sulfate. CYP11B2 was absent in the adrenal tumors, but was positive in the extra-tumor area as aldosterone-producing cell cluster (arrows). Scale bars represent (**a**) 10 mm, (**b**) 300 μm, (**c**)-(**e**) 6 mm, (**f**) 7 mm, and (**g**) 6 mm. CYP, cytochrome P450; HSD, hydroxysteroid dehydrogenase; CYP, cytochrome P450; HSD3B, 3β-hydroxy-Δ5-steroid dehydrogenase.

Thus, the patient was diagnosed with SCS [10] due to bilateral functioning autonomous cortisol secreting adrenal tumors [11]. Although serum cortisol and urinary free cortisol levels decreased after left unilateral laparoscopic adrenalectomy, the paradoxical response persisted (Table 2). Pathological examination revealed adrenocortical adenoma. The tumor consisted of round to polygonal-shaped cells with microvascular or eosinophilic cytoplasm, proliferating in an alveolar fashion, accompanied by hemorrhage, inflammatory infiltrate and lipochrome deposit, leading to the diagnosis of adrenal adenoma. Immunohistochemical analysis showed positive expression for cytochrome P450 (CYP) 17A1, HSD3B type-1, HSD3B type-2, dehydroepiandrosterone sulfotransferase, and CYP11B1, but not for CYP11B2 (Fig. 3). Genetic examination of the adrenal tumor revealed the somatic *GNAS* mutation p.R201H, which is known to be responsible for McCune-Albright syndrome, although sporadic GNAS mutations have also been reported [2]. No *PRKAR1A* mutation was detected in either the adrenal adenoma or the peripheral blood. The patient was treated with eplerenone, which had successfully ameliorated persistent hypertension and hypokalemia at her one year follow up visit.

## Genetic analysis

Genomic DNA was extracted from fresh frozen adenoma tumor tissues and peripheral blood. Sureselect Human All Exon V6 (Agilent Technologies, Santa Clara, CA, USA) was used for exome capture followed by massive parallel sequencing on the Illumina platform (HiSeq2500; Illumina, San Diego, CA, USA). Sequence alignment and mutation calling were performed using our in-house pipeline, as previously described [2, 14] (Mean depth: 134.6 and 131.1). Candidate mutations for somatic mutations were filtered using the following criteria: (i) strand ratio ≠ 0,1, (ii) number of variant reads in tumor sample ≥ 4, (iii) number of variant reads in normal sample ≤ 1, (iv) Fisher’s exact *p* < 0.1, (v) EBCall *p* value < 10^− 4^, (vi) variant allele frequency (VAF) in tumor sample ≥ 0.05, (vii) annotated in exonic or splicing areas.

Candidate germline mutations with (i) strand ratio ≠ 0,1, (ii) VAF between 0.4 and 0.6, (iii) number of variant reads ≥4, (iv) EBCall p value < 10^− 4^ were further filtered by excluding synonymous variants and known variants with frequency of ≥0.1% in 1000 Genomes Project (Nov. 2010 release), Exome Sequencing Project (ESP6500), and the Human Genome Variation Database (HGVD; October 2013 release).

Copy number analysis was performed using our in-house pipelines, CNACS [15], which could identify the copy number alterations (CNAs) using pooled normal samples as a reference.

As a result, we identified somatic *GNAS* p.R201H as the driver mutation of SCS (VAF: 0.379). No other somatic/germline mutations or CNAs was detected in any known causative genes including *PRKAR1A*.

## Discussion and conclusion

This is the first reported case of SCS [10] due to autonomous cortisol producing adrenal adenoma [11] harboring a *GNAS* mutation, which exhibited the paradoxical cortisol response reported in patients with PPNAD. On immunohistochemical examinations, the resected adrenal adenoma showed potentials to produce cortisol and DHEA-sulfate, but not aldosterone. *GNAS* mutation-harboring Cushing’s syndrome may provide clues to elucidate the mechanisms by which the glucocorticoid receptor (GR)-regulated steroidogenic enzymes operate in the adrenal gland, and the pathophysiology of SCS with extra-adrenal tumors.

Paradoxical cortisol response may provide a clue to predicting various mutations that enhance the cAMP-dependent PKA pathway, and to disclose the mechanisms which lead to the development of this type of Cushing’s syndrome. Most cortisol-producing adrenocortical adenomas with paradoxical response reportedly harbor somatic *PRKAR1A* mutations [7, 16]. However, in a previous study of patients with Cushing’s syndrome, harboring somatic catalytic α subunit of PKA (*PRKACA*) or *GNAS* mutation, 17/20 patients showed higher serum cortisol levels when treated with 8 mg dexamethasone, but not with 1 mg of dexamethasone (Table 2) [2], which is consistent with the present case. Thus, patients who exhibit high cortisol levels in response to high-dose dexamethasone might show paradoxical response. Furthermore, mildly elevated urinary free cortisol levels are detected when Liddle’s test is performed in patients with ACTH-independent macronodular adrenal hyperplasia and/or adrenocortical cortisol-producing adenoma [17]. These patients present with aberrant expression and/or mutations of G protein-coupled receptors, both of which could enhance the cAMP-dependent PKA pathway [17]. In patients with PPNAD, the overexpression of GR in affected adrenal nodules [8] and the role of GR-stimulation in paradoxical response to dexamethasone [9] have been reported previously. *GNAS* mutations activate PKA via activating the cAMP-dependent signaling pathway, and subsequently enhance GR-dependent positive cortisol synthesis, which might lead to autonomous cortisol production [9].

The present case demonstrated the potential of the adrenal adenoma with *GNAS* mutation to produce cortisol and DHEA-sulfate, but not aldosterone. Immunohistochemical studies revealed that the adrenocortical adenoma in the present case expressed several steroidogenic enzymes including CYP 11B1, a cortisol synthetase (Fig. 3c-g), but not CYP11B2, an aldosterone synthetase (Fig. 3h). The presence of aldosterone-producing cell clusters suggested that the extra-tumor over-secretion was responsible for mild primary aldosteronism in the present case [18], although we did not look for *GNAS* mutation in the extra-tumor lesion in the adrenal cortex. Although the adrenal adenoma in this patient did not produce aldosterone, *GNAS* mutations have been detected in adrenocortical adenomas producing both aldosterone and cortisol, the aldosterone-producing ability in these tumors has not been confirmed immunohistochemically [19] (Fig. 3h). However, the tumor was positive for DHEA-sulfotransferase (Fig. 3f), which could possibly explain the high-normal levels of DHEA-sulfate. Thus, it can be concluded that *GNAS* mutations may activate all steroidogenic enzymes except for those involved in aldosterone production. Our results suggest that cAMP-dependent PKA pathway is responsible for the production of androgen but not aldosterone in adrenal tumors.

Cushing’s syndrome with paradoxical response to dexamethasone is known to present with a severe phenotype [9], because adrenocortical cells with cAMP-PKA signaling pathway mutation exhibit higher hormonal productivity [2]. Nonetheless, a mild form of Cushing’s syndrome was reported in a case of ACTH-independent macronodular adrenal hyperplasia due to *GNAS* mutation [17]. Although we could not conclusively explain the reason for SCS in the present case, one plausible explanation could be the mosaic feature of mutated lesions [20], similar to the main genetic mechanism in McCune-Albright syndrome. Hence, further investigation of patients with McCune Albright syndrome, who had *GNAS* mutation-harboring Cushing’s syndrome, could provide a better understanding of the pathophysiology of this type of Cushing’s syndrome, which might even be considered as a partial form of McCune Albright syndrome [12, 21].

Various tumors were observed in the present case (Fig. 2). In a large case series study of patients with McCune-Albright syndrome [22], the prevalence of *GNAS* mutation-harboring Cushing’s syndrome due to functional adrenal tumors was reported at 7.1%. In another study a high prevalence of extra-adrenal malignancy was reported in patients with functioning bilateral adrenal tumors [23], with tumors being dispersed throughout the body, but the locations were similar to those in the present case, namely endometrium, breast, thyroid gland, and colon. Since we did not detect *GNAS* mutation in any other tissue, we could not conclude whether this case was a partial form of McCune Albright syndrome or a sporadic case of *GNAS* mutation identified in the adrenal adenoma [2, 12, 21]. On the other hand, enhanced signaling of the cAMP-PKA pathway due to *GNAS* and *PRKAR1A* mutations, leads to the activation of the Wnt/β-catenin signaling pathway that might explain the pattern of endocrinological dysregulations and tumorigenesis in this case [12]. Our findings in the present case suggested that the activation of the cAMP-dependent PKA pathway in bilateral adrenocortical adenomas with paradoxical cortisol response and *GNAS* mutation, may result in multiple extra-adrenal neoplasms and malignancies [5, 24].

Additional cases would be required to support our results. In addition, there were a few limitations for genetic analysis in this study: WES and WES-based copy number analysis often miss structure variants and micro deletion/amplification. Due to this limitation, we might have missed germline mutations in some genes including *PRKAR1A*. Additional analysis with other platforms or sequencing other tumor tissues in this patient might help reach a comprehensive understanding of the molecular mechanisms in this kind of SCS with paradoxical cortisol response to oral dexamethasone and multifocal tumorigenesis.

In conclusion, this is the first reported case of SCS due to an autonomous cortisol secreting adrenal adenoma, harboring a *GNAS* mutation, which exhibited a paradoxical increase in urinary free cortisol levels in response to the oral administration of dexamethasone. This type of paradoxical response may provide a unique clue in the diagnosis of various types of cAMP-dependent PKA pathway-related Cushing’s syndrome, including *GNAS* mutations, as the *GNAS* mutation-harboring adrenal tumor in this case showed potentials to produce cortisol and DHEA-sulfate, but not aldosterone. The possibility of *GNAS* mutations should be considered in patients with functional bilateral adrenal tumors to detect curable malignancies and contribute to better prognosis.

## Abbreviations

cAMP: Cyclic adenosine monophosphate
CNAs: Copy number alterations
CYP: Cytochrome P450
DHEA: Dehydroepiandrosterone
GNAS: Stimulatory G protein-α subunit
GR: Glucocorticoid receptor
PAC: Plasma aldosterone concentration
PKA: Protein kinase A
PPNAD: Pigmented nodular adrenocortical disease
PRA: Plasma renin activity
PRKACA: Catalytic alpha subunit of protein kinase A
PRKAR1A: Protein kinase type 1α regulatory subunit
SCS: Subclinical Cushing’s syndrome
VAF: Variant allele frequency
